# Effects of Venous Superdrainage and Arterial Supercharging on Dorsal Perforator Flap in a Rat Model

**DOI:** 10.1371/journal.pone.0160942

**Published:** 2016-08-11

**Authors:** Jun Zheng, Shanshan Xi, Maochao Ding, Hong Li, Wei Xu, Maolin Tang, Shixin Chen

**Affiliations:** Department of human anatomy, Wenzhou Medical University, Wenzhou, China; Johannes Kepler University Linz, AUSTRIA

## Abstract

**Objective:**

To comparatively assess the effects of venous superdrainage and arterial supercharging on dorsal perforator flap survival.

**Materials and Methods:**

Sixty male Sprague-Dawley rats (450–550g) were randomly divided into three groups (n = 20), including control group (Control) and experimental groups A (venous superdrainage, Exp. A) and B (arterial supercharging, Exp. B). At postoperative day 7, survival areas of the flaps were evaluated and all animals underwent angiography. Laser Doppler was used to evaluate flap perfusion from 0h to 7days after surgery. Histology with hematoxylin and eosin staining was used to count microvessels. Tissue of “Choke vessels”was excised for quantification of hypoxia inducible factor-1α (HIF-1α) and vascular endothelial growth factor (VEGF) by western blot assay at 6h and 7days after surgery.

**Results:**

In the Exp. A group, almost all flaps survived (98.2±1.6%); in the Exp. B and control group, survival areas accounted for 78.8±8.5% and 60.3±7.8%, respectively (P <0.001). In addition, Exp. A animals showed improved *anastomosis* of choke vessels 2 compared with the Exp. B and Control groups. Furthermore, flap blood flow and *partial pressure of oxygen* in the Exp. A group were significantly higher compared with values obtained for the Exp. B and Control groups, from 6 hours to 7 days after surgery. *More microvessels* were found in the Exp. A group *(11*.*65±1*.*33) than in* Exp. B (*9*.*25±0*.*34)* and control (*7*.*25±0*.*91) animals on POD 7*. The relative expression level of HIF-1α and VEGF were significant at 6h and 7days after surgery.

**Conclusions:**

Venous superdrainage in rat dorsal perforator flap is more effective than arterial supercharging in promoting flap survival, and could effectively alter hemodynamics in the microcirculation and stimulate blood vessel formation.

## Introduction

In the clinic, it is not uncommon for skin flaps to undergo necrosis after transplantation. Multiple experimental studies have shown that arterial inflow results in arterial augmentation and reduced flap necrosis [1–7]. Interestingly, some studies suggested that venous drainage can be highly effective in increasing survival of skin flaps; this can be done by venous superdrainage (using an additional vein to increase drainage) or arterial supercharging (adding an artery) [8–15]. Although reports assessing venous superdrainage and arterial supercharging are countless, there are still many unresolved problems in the clinic. For example, whether hemodynamics in the microcirculation after venous superdrainage and arterial supercharging have different effects on improving flap survival is unknown. In addition, it is unclear whether systemic vascular resistance in venous superdrainage and arterial supercharging have different effects on flap survival. Finally, the question whether venous superdrainage could effectively stimulate blood vessel formation to improve flap survival remains unanswered. The purpose of this study was to comprehensively assess the effects of venous superdrainage and arterial supercharging on flap survival and neoangiogenesis.

## Methods

### Surgical technique

**Fig 1:**
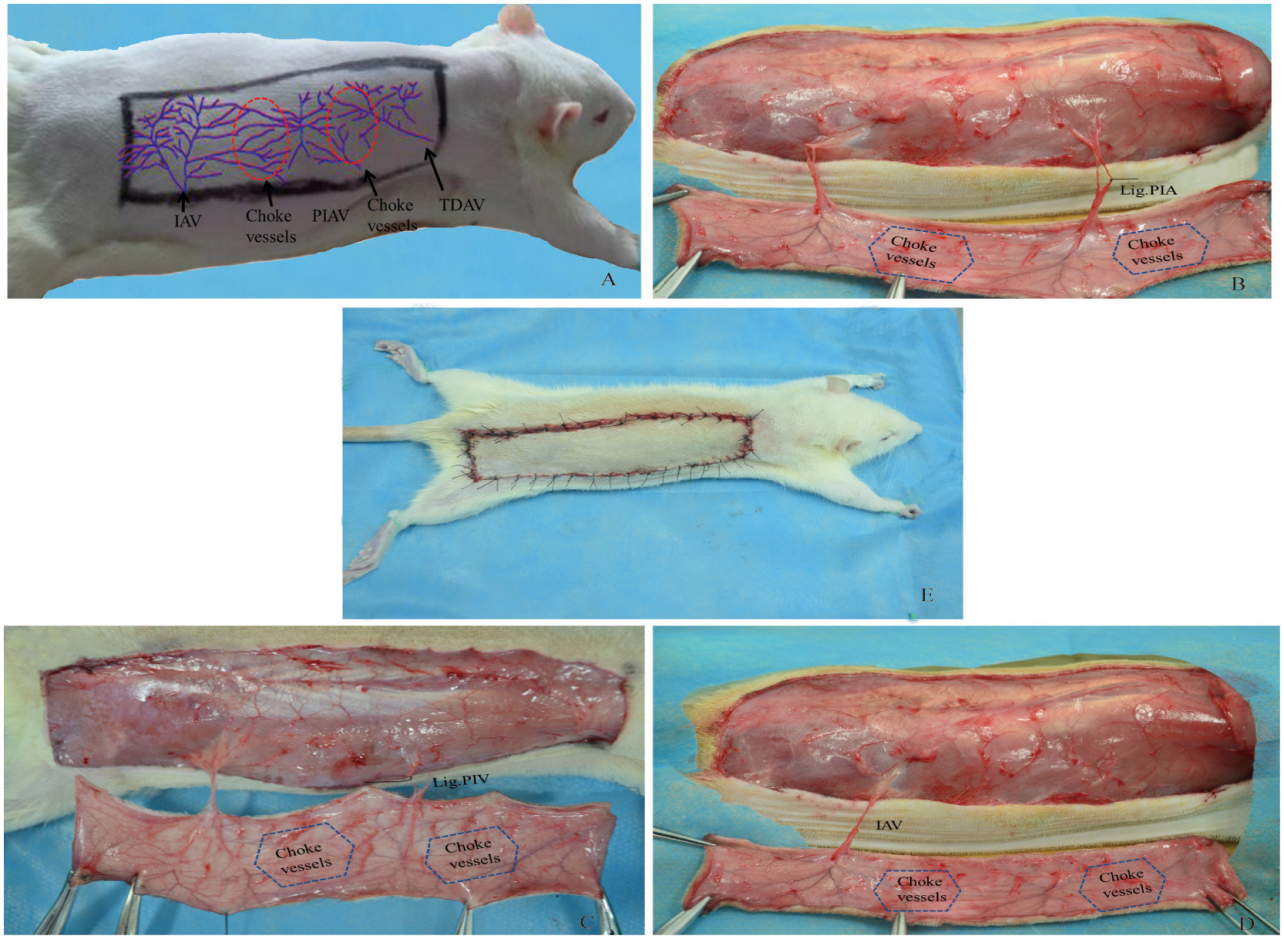
Skin flap design and surgical procedure. Right dorsal perforator flap model measured 3×12 cm (Fig 1A), Experimental group A, posterior intercostal artery (PIA) was ligated while accompanying vein was preserved (Fig 1B); Experimental group B, posterior intercostal vein (PIV) was ligated while accompanying artery was preserved (Fig 1C); Control group, posterior intercostal artery and vein were ligated, only vascular pedicle (iliolumbar artery and vein, IAV) was preserved (Fig 1D). Finally, the flap was sutured back into its location (Fig 1E). “Choke vessels”: anastomosis area of perforator vessels, IAV: iliolumbar artery and vein, PIAV: posterior intercostal artery and vein, TDAV: thoracodorsal artery and vein.

**Fig 2:**
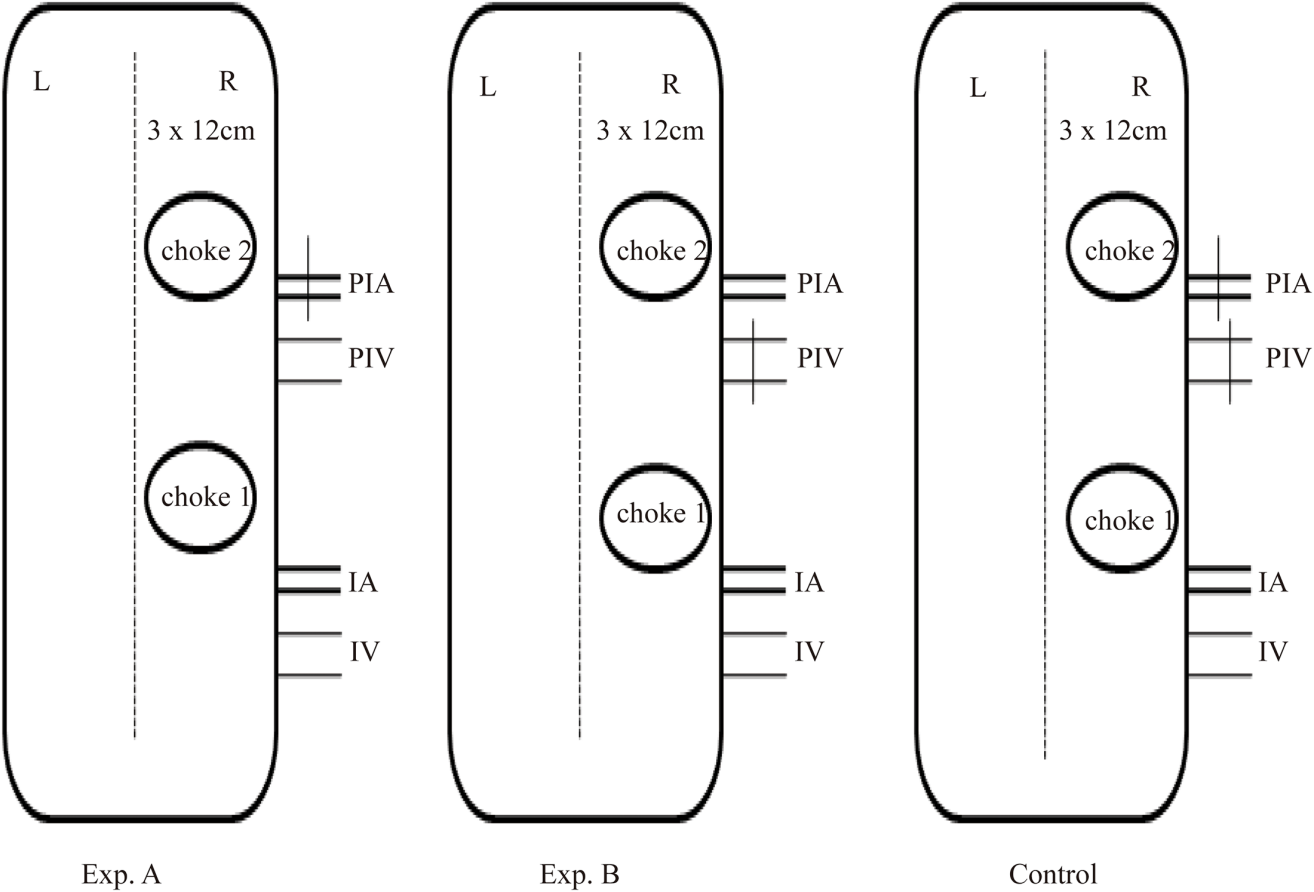
The schematic drawing of grouping.

The animals were purchased from the Wenzhou Medical University Center for Laboratory Animals (license no. ZJ2014-034). This study was approved by the Committee of Animal Care and Use for Research and Education (CACURE) of Wenzhou Medical University and performed in accordance with the National Institutes of Health Guidelines for the Care and Use of Laboratory Animals. Sixty male Sprague-Dawley rats (450–550g) were randomly divided into three groups (n = 20), including control group (Control) and experimental groups A (venous superdrainage, Exp. A) and B (arterial supercharging, Exp. B). Ten additional rats served as a normal group. All surgical instruments were autoclaved before use. After anaesthesia with 5% chloral hydrate (intraperitoneal injection, 0.6 mL/100g), the animals were shaved over the entire back, and placed in the prone position. Flap boundaries were based on anatomical landmarks: ① midline of the back; ② cranial border, 1 cm below the right shoulder blade; ③ caudal border, at the level of the right iliac crest. Flap size was approximately 3×12 cm (Fig 1A). A sterile towel was then placed onto the back, and the operational area disinfected with iodine and 75% alcohol. The flap was then elevated on the animal’s back. The two and a half angiosomes, i.e. the posterior intercostal perforator vessel, the iliolumbar perforator vessel, and the distal part of the thoracodorsal perforator vessel [16,17], were easily identified after superficial fascia removal. One and a half choke vessels were found between the angiosomes. In the Exp. A group, the posterior intercostal artery was isolated and ligatured, preserving the accompanying vein (Fig 1B). In the Exp. B group, the posterior intercostal vein was ligatured while the accompanying artery was preserved (Fig 1C). The iliolumbar vessel was used as a vascular pedicle in both experimental groups. In controls, only the iliolumbar vessel was used as a pedicle, and the posterior intercostal perforator vein and artery were ligatured (Fig 1D). Finally, the flap was sutured back into its location (Fig 1E). A schematic drawing of the entire procedure is shown in Fig 2. Postoperatively, animals received analgesic treatment with metamizole (5 mg/kg, intramuscular injection, china).

### Flap survival assessment

At POD 7, flaps were photographed using a digital camera (Canon, Tokyo, Japan). Images were imported into Image J (Image Processing and Analysis in Java, National Institutes of Health, Bethesda, MD, USA), which was used to assess flap survival areas.

### Perforator flap angiography

To assess blood supply to the flap at POD 7, all animals were anaesthetized with 5% chloral hydrate (intraperitoneal injection, 0.6 mL/100g). After injection with low-molecular-weight heparin sodium via the lateral tail vein (5000 IU, Bo Yun Biotechnology Company, Shanghai, China), the animals were euthanized with an overdose of chloral hydrate for 10 min. This was followed by perfusion of 5% lead oxide-gelatin into the left common carotid artery, until the animal limbs turned red. Rat cadavers were then incubated at 40℃ in a water bath (Ningbo Tianheng Instrument, SC-15, Ningbo, China) for 5 h [18], followed by overnight freezing. Skin was dissected carefully along the abdominal midline at the level of the deep fascia. After dissection, skin specimens were spread out and flattened for radiography on a computerized radiography system (XG-1, Fujifilm, Tokyo, Japan), to assess changes in the two vascular territories and choke vessels.

### Flap perfusion and transcutaneous oxygen pressure

Rats were anaesthetized at 0 h, 6 h, 1 day, 2 days, 3 days and 7 days after surgery to monitor the degree of flap blood perfusion and transcutaneous oxygen pressure (TcpO_2_) by laser Doppler (PeriFlux System 5000, PERIMED, Sweden). The flap perfusion probe was placed in the anastomosed zone *(*“choke zone”*)* and scanned for 2 min after stabilization; blood flow was expressed as perfusion units. TcpO_2_ detection was carried out with the probe also in the “choke zone” to detect TcpO_2_, by scanning for 10 min; results were expressed as TcpO_2_ units.

### Microvessel count

At POD 7, the *“*choke vessels 1*”* tissue was excised for histological staining. Specimens were cut into 1×1 cm pieces, sectioned into 5μm slices, and prepared for hematoxylin and eosin staining. Microvessels were counted under a light microscope to determine the microvascular density of the flap. Areas of high microvessel were scanned at low power (40x total magnification) and microvessel counts were carried out in high power (200x) fields. A single endothelial cell and dimmed microvessels were not considered.

### Western blot

The tissues of “choke vessels 1” were lysed for extraction of total cellular protein using tissue extraction kit (Biovision Corporation, California, USA). The proteins were processed using sodium dodecyl sulphate–polyacrylamide gel (Sigma-Aldrich, Missouri, USA) electrophoresis and transferred on to a nitrocellulose filter membrane (Pall corporation, New York, USA). The membrane was blocked with 5% skim milk (Becton, Dickinson and Company, New Jersey, USA) for 2 h and incubated sequentially with monoclonal rabbit anti-rat primary antibodies against HIF-1*α* (1:1000, Abcam, USA), VEGF (1:1000, Abcam, USA) and β-actin (1:1000, Santa Cruz Biotechnology), and then probed with goat anti-rabbit secondary antibody. Antibody-binding bands were detected using enhanced chemiluminescence plus reagent (Advansta Corporation, California, USA). Intensities of target bands relative to β-actin were analyzed using Image J (Image Processing and Analysis in Java, National Institutes of Health) to calculate the gray values. The experiment was independently repeated three times.

### Statistical analysis

Statistical analyses were carried out with the SPSS software for Windows (version 17.0; SPSS Inc., Chicago, USA). Data are mean±standard deviation (SD), and were compared using Bonferroni test of one-way ANOVA and Mann-Whiteney U test. Two-tailed *P*<0.05 was considered statistically significant.

## Results

**Fig 3:**
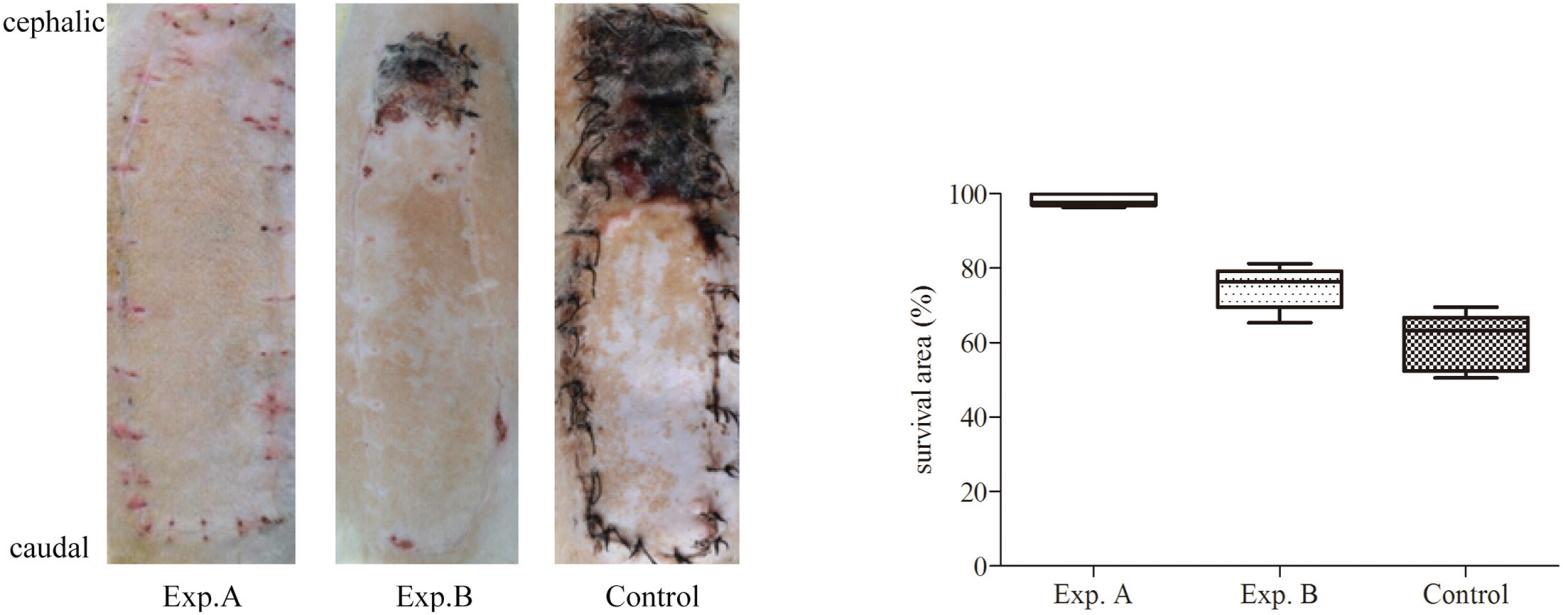
Flap survival. Comparison of the flap survival, survival area was showed as means±SD.

Venous superdrainage in rat dorsal perforator flap was efficacious. At POD 7, almost all flaps in the Exp. A group survived (98.2±1.65% survival); in the Exp. B group, survival areas amounted to 78.8± 8.5%; in control animals, the distal portion of the flap showed necrosis, with survival areas amounting to 60.3±7.8%. Clearly, survival areas in Exp. A animals were significantly larger than those of Exp. B (*P* = 0.016). Survival areas in both Exp. A and Exp. B groups were significantly increased compared with control values (*P* < 0.001) (Fig 3).

**Fig 4:**
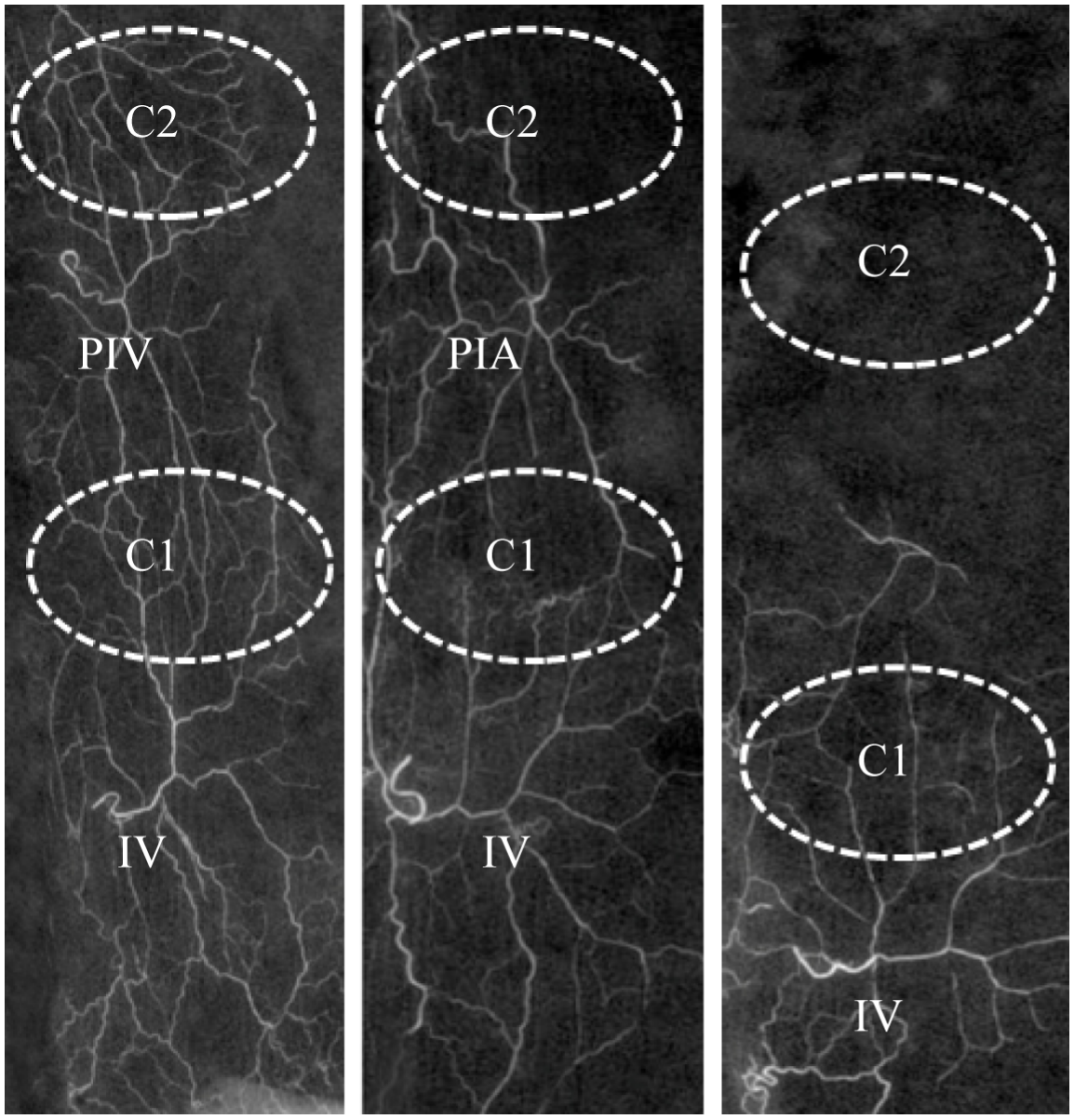
Angiography in each group. White dashed circles represent choke vessels, IV: iliolumbar vein, PIV: posterior intercostal vein, PIA: posterior intercostals artery, C1: “choke vessels 1”, C2: “choke vessels 2”.

At POD 7, according to angiography data, the posterior intercostal vessel and the distal part of the thoracodorsal perforator vessel were connected by choke vessels. In addition, Exp. A animals showed improved anastomosis of choke vessels 2 compared with the Exp. B and Control groups. In the Control group, because of venous congestion, vascular anastomosis and distal venosome were difficult to identify (Fig 4).

**Fig 5:**
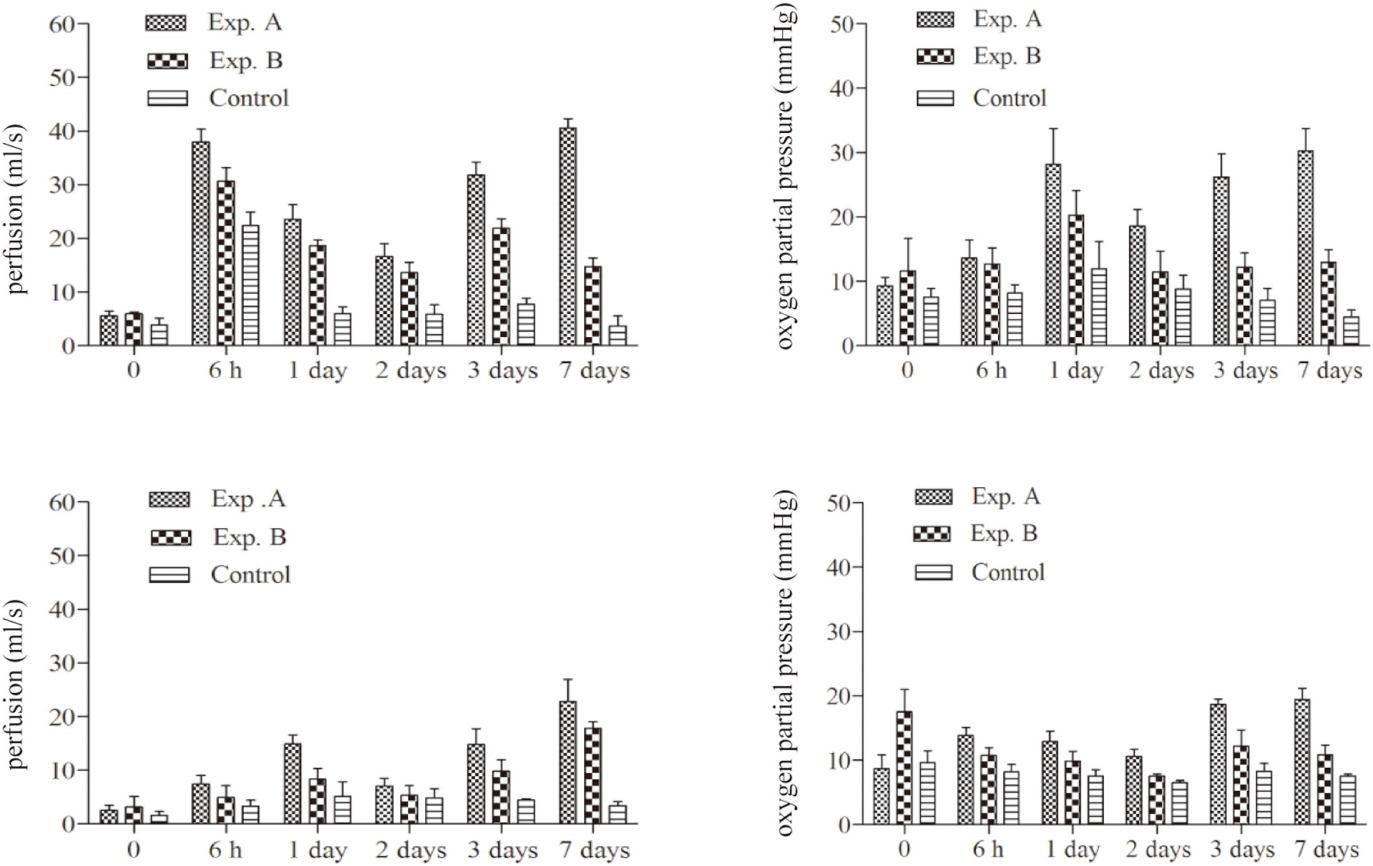
The comparison of flap perfusion and partial pressure of oxygen from 6 h to 7 days postoperatively. Left: perfusion of “choke vessels 1” and “choke vessels 2”. Right: oxygen partial pressure of “choke vessels 1” and “choke vessels 2”.

Flap blood flow and partial pressure of oxygen increased in all groups from 6 hours to 7 days after surgery. Blood flow of “choke vessel 1” in the Exp. A group was significantly higher than Exp. B and control values, from 6 hours to 7 days post-operation. In addition, transcutaneous oxygen pressure (TcpO_2_) of “choke vessel 1” was significantly increased in Exp. A animals compared with the values obtained for the Exp. B and Control groups, from 6 hours to 7 days post-operation. Meanwhile, blood flow and partial pressure of oxygen of “choke vessel 1” were higher than those of “choke vessel 2”(all *P*<0.05)as shown in Fig 5.

More microvessels were found in Exp. A animals (11.65±1.33) compared with the Exp. B (9.25±0.34) and Control (7.25±0.91) groups at POD 7 (Table 1) (*P*<0.001).

**Table 1:** Average microvessel count in each group (AMC) at POD 7.

groups	n	AMC(m±SD)
Exp.A	10	11.65±1.33
Exp.B	10	9.25±0.34
Control	10	7.25±0.91

There was statistically significant difference in the number of microvessels between Exp. A and Exp. B (*P* = 0.018), differences also were noted between Exp. A and control (*P*<0.001).

Protein expression was verified as the ratio of the gray values of the target protein to β-actin (mean±SD) at 6h and 7days post-operation. At 6h post-operation, HIF-1α expression increased in all groups as compared to the normal group (*P* = 0.003). As compared to the Exp. B and control groups, VEGF expression in the Exp. A group was higher (*P* = 0.006). HIF-1α and VEGF expression levels were significantly different on day 7 post-operation (all *P*<0.05; S1 Fig).

## Discussion

Since 1984, cases of effective venous drainage promoting flap survival have increased [10–13,19]. In experimental animals, many studies have used abdominal models based on the superficial inferior epigastric vessel or deep inferior epigastric perforator vessel to reduce congestion and improve flap survival rate by venous drainage [10,11,14]. We chose the dorsal instead of abdominal perforator flap, since it is thin and easy to hide if used in clinic [20]. Multiple experimental studies showed arterial inflow results in arterial augmentation and improved flap survival [1–3,21]. However, how do supercharging and superdrainage ancillary maneuvers increase perforator flap survival? Interestingly, improved flap survival was accompanied by better blood supply and increased microvascular density [22,23]. In the present study, venous superdrainage resulted in higher pedicle flap survival, compared with arterial supercharging. In addition, blood perfusion and transcutaneous oxygen pressure were higher after venous superdrainage compared with arterial supercharging and control groups. It is possible that blood supply was lower in Exp. B and Control groups compared with that in the Exp. A group. Indeed, blood flow is associated with the resistance of vessels and hemodynamic remodeling [8, 24]. In Exp. A, the arterial input from the lumbar vessels could have been sufficient to vascularise the entire flap from the arterial input and hereby create enough retrograde pressure in the venous system to push the blood back into the posterior intercostal vein and the lumbar vein. In Exp. B, the anterior input of two arteries would not necessarily increase the anterograde pressure in the capillary system, but the occlusion of the intercostal vein might have created a situation where the retrograde pressure in the venous system of the most distal part of the flap was insufficient to push back the venous blood all the way down to the lumbar vein. Obviously in the control group, the anterograde pressure would have been sufficient and the retrograde pressure in the venous system would have been insufficient (S2 Fig). Therefore, hemodynamic remodeling has an effect on survival of the flap. On the other hand, hypoxia and vascular endothelial growth factor (VEGF) stimulate angiogenesis [25,26]. Our findings in this study provide evidence for this theory. VEGF protein levels were higher in “choke zone 1” of the Exp. A compared with the Exp. B and the Control, which indicate mild hypoxia can induce VEGF expression (S1 Fig). The number of microvessels increased is a result of increased VEGF expression. These also verified the success of venous superdrainage with a great advantage compared with that of arterial supercharging.

Choke vessels are blood vessels whose calibers gradually decrease, and connected with tiny blood vessels; the main portion of the systemic resistance network in flaps may be located in these choke zones [27]. We used Lead oxide-gelatin imaging to observe choke vessels, as a classic and simple way to assess vascular morphology [18,28]. At POD 7, in terms of angiography, improved anastomosis of choke vessels and higher microvessel counts were obtained in the Exp. A group compared with the Exp. B and Control groups. However, in control animals, distal angiosomes were difficult to identify because of necrosis. More choke vessels anastomosing adjacent angiosomes after venous superdrainage was consistent with the high blood perfusion rate observed by laser Doppler imaging. Choke veins promoting blood outflow from the perforator vein and reducing congestion corroborates previous findings [29–31]. The primary purpose of this study was to investigate how venous superdrainage and arterial supercharging improve flap survival. The main limitations of this model are: (1) rats need to be anesthetized many times when monitoring perfusion; (2) the dynamic change of blood vessels cannot be observed *in vivo*. This is still largely a descriptive study. Quantitative studies analyzing diameter changes, and differences in inflammatory response, and protein and cytokine levels in rats of different ages have yet to be conducted. In addition, the mechanisms underlying the distinct effects of venous superdrainage and arterial supercharging require further assessment.

## Conclusions

The effect of venous superdrainage is more apparent than arterial supercharging, can effectively alter hemodynamics of microcirculation, especially in the “choke zone”. Effectively maintenance of venous superdrainage had more enduring resistance for hypoxia and ischemia. Flap transplantation could therefore be clinically easier and more reliable when making use of venous drainage to change microcirculation during surgery. Although its regulatory mechanism and signal transduction pathways in microcirculation require further study.

## Supporting Information

S1 Fig Protein expression. Protein expression level was verified as the ratio of the gray values of the target protein to β-actin (mean±SD) at 6h (left) and 7days post-operation (right).
(TIF)

S2 Fig Schematic of blood flow in a multiple vascular territory flap before and after surgery. **(A)** Perforator vascular territory and vascular networks of choke zone in normal physiological conditions. **(B,C,D)** Hemodynamic remodeling after surgery. Black dashed circles represent “choke vessels”. Black arrows show venous inflow, and red arrows represent arterial outflow.
(TIF)
