# Supplementary figures and images for: Effects of Venous Superdrainage and Arterial Supercharging on Dorsal Perforator Flap in a Rat Model
Source: PLoS One. 2016 Aug 11;11(8):e0160942. doi: 10.1371/journal.pone.0160942 (PMC4981354; doi:10.1371/journal.pone.0160942)

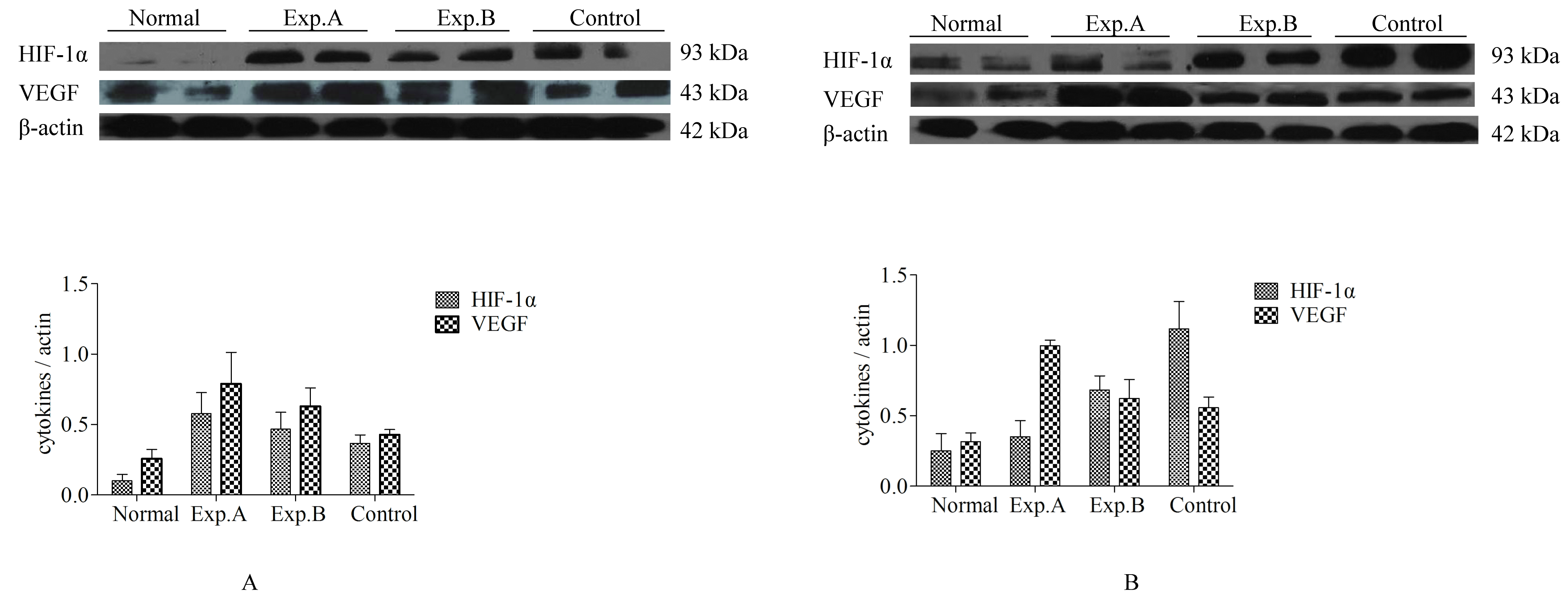

Supplement: S1 Fig — Protein expression level was verified as the ratio of the gray values of the target protein to β-actin (mean±SD) at 6h (left) and 7days post-operation (right). (TIF) [file pone.0160942.s001.tif]

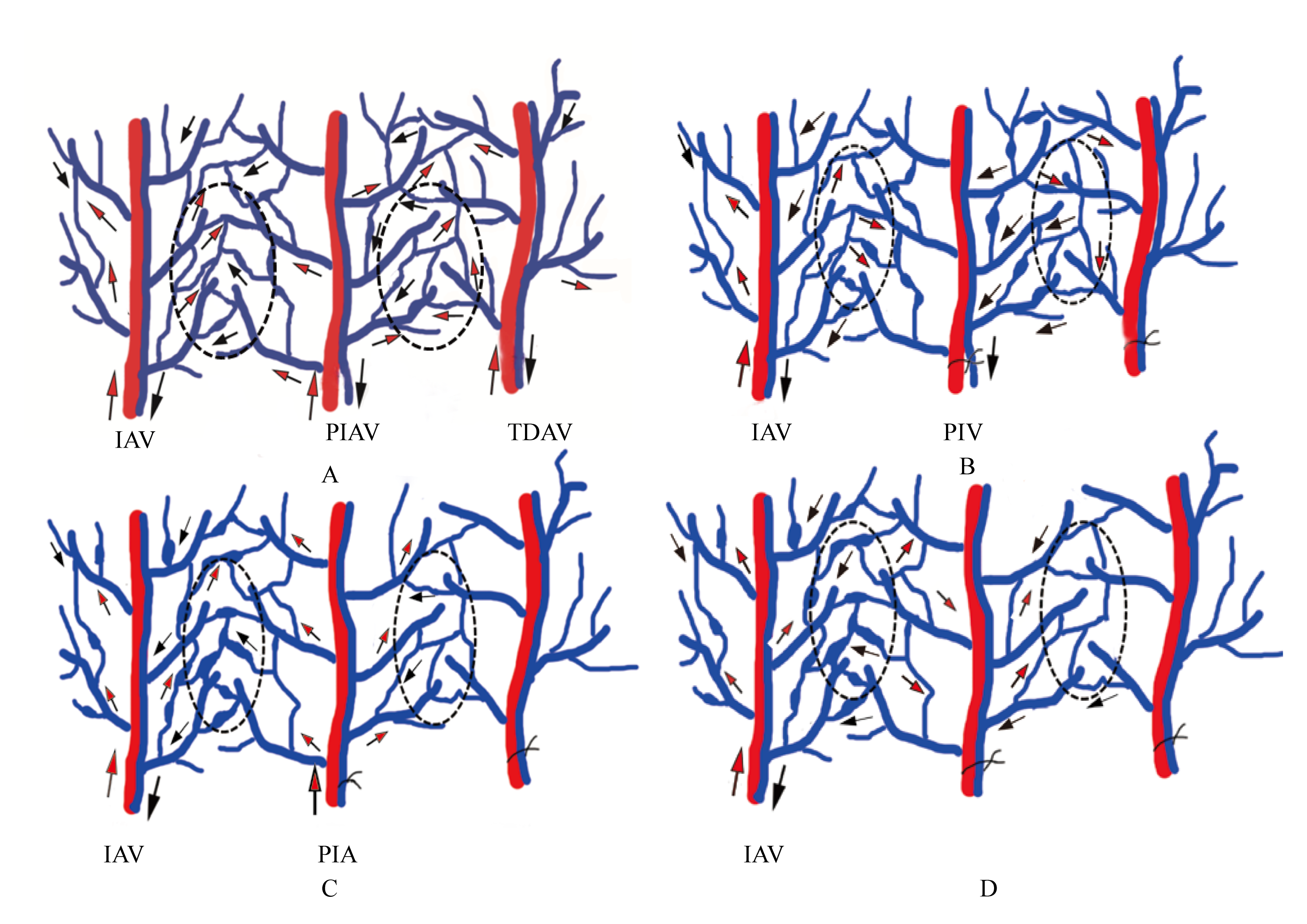

Supplement: S2 Fig — (A) Perforator vascular territory and vascular networks of choke zone in normal physiological conditions. (B,C,D) Hemodynamic remodeling after surgery. Black dashed circles represent “choke vessels”. Black arrows show venous inflow, and red arrows represent arterial outflow. (TIF) [file pone.0160942.s002.tif]
